# Supplementary material for: 12-month substance use disorders among first-year university students in Kenya
Source: PLoS One. 2023 Nov 27;18(11):e0294143. doi: 10.1371/journal.pone.0294143 (PMC10681253; doi:10.1371/journal.pone.0294143)
Supplement: Attachment — Submitted filename: 2nd Response to reviewers comments College Survey Jaguga.docx [file pone.0294143.s004.docx]

Dr. Florence Jaguga

Moi Teaching & Referral Hospital

P.O. BOX 3-30100

**ELDORET, KENYA**

15^th^ October 2023

Emily Chennette

Editor-in-chief

Plos One

Dear Madam,

**SUBMISSION OF REVISED MANUSCRIPT AND RESPONSE TO REVIEWERS’ COMMENTS**

We wish to submit a revision of the manuscript entitled “**12-month substance use disorders among first-year university students in Kenya”** for consideration by the Plos One journal.

A point-by-point response to the reviewers’ and editor’s comments is attached to this letter.

Thank you for your consideration. We look forward to a favorable response.

Sincerely,

**Florence Jaguga (MBchB, MMed Psychiatry)**

**Response to reviewers’ and Editor’s comments**

The authors thank the Editor and reviewers for the comments.

A point-by-point response to the comments has been provided below.

**Reviewer #1: Did you check with Harvard about the sharing of data? I still don't think you can publically share but can note that.**

Yes. We did check with Harvard, and they confirmed that since this paper, wasn’t based on cross-national data, but just Kenyan data, it was up to us to decide whether to make the data available or not. The authors have therefore decided to make the data available.

**Comment: Check English and grammer throughout manuscript - still reference to 'we' e.g. line 94, 136, 185, 262, 292, 303, 333, 335**

Response: The authors have restructured the sentences to remove ‘we’ and ensure clarity throughout the document.

**Comment: Note the tables need to be formatted correctly - no vertical lines**

Response: The vertical lines have been removed on all tables in the results section

**Comment: In response to reviewer 2 it might be good to include something like the following:
Good concordance has been found between the WMH-CIDI and clinical assessments
Haro JM, Arbabzadeh-Bouchez S, Brugha TS, de Girolamo G, Guyer ME, Jin R, et al. Concordance of the Composite International Diagnostic Interview Version 3.0 (CIDI 3.0) with standardized clinical assessments in the WHO World Mental Health surveys. International journal of methods in psychiatric research. 2006; 15(4):167–80. PMID: 17266013**

Response: Thank you for this comment. We have revised the response to reviewer 2 to read as follows**:**

The tool used for the World Mental Health International College Student (WMH-ICS) Initiative is designed to generate accurate epidemiological data on mental, substance, and behavioral disorders among college students. Other studies conducted as part of the initiative have reported on epidemiology of disorders. The authors prefer to keep the diagnoses as disorders in this manuscript. (<https://www.hcp.med.harvard.edu/wmh/college_student_survey.php>).

In addition, good concordance has been found between the WMH-CIDI and clinical assessments.

(Haro JM, Arbabzadeh-Bouchez S, Brugha TS, de Girolamo G, Guyer ME, Jin R, et al. Concordance of the Composite International Diagnostic Interview Version 3.0 (CIDI 3.0) with standardized clinical assessments in the WHO World Mental Health surveys. International journal of methods in psychiatric research. 2006; 15(4):167–80. PMID: 17266013)
